# EVOLVING FRIENDSHIPS AND SHIFTING ETHICAL DILEMMAS: FIELDWORKERS' EXPERIENCES IN A SHORT TERM COMMUNITY BASED STUDY IN KENYA

**DOI:** 10.1111/dewb.12009

**Published:** 2013-02-21

**Authors:** Dorcas M Kamuya, Sally J Theobald, Patrick K Munywoki, Dorothy Koech, Wenzel P Geissler, Sassy C Molyneux

**Keywords:** field workers, research ethics, social relations, developing countries, benefit sharing, consent processes, household studies

## Abstract

Fieldworkers (FWs) are community members employed by research teams to support access to participants, address language barriers, and advise on culturally appropriate research conduct. The critical role that FWs play in studies, and the range of practical and ethical dilemmas associated with their involvement, is increasingly recognised. In this paper, we draw on qualitative observation and interview data collected alongside a six month basic science study which involved a team of FWs regularly visiting 47 participating households in their homes. The qualitative study documented how relationships between field workers and research participants were initiated, developed and evolved over the course of the study, the shifting dilemmas FWs faced and how they handled them. Even in this one case study, we see how the complex and evolving relationships between fieldworkers and study participants had important implications for consent processes, access to benefits and mutual understanding and trust. While the precise issues that FWs face are likely to depend on the type of research and the context in which that research is being conducted, we argue that appropriate support for field workers is a key requirement to strengthen ethical research practice and for the long term sustainability of research programmes.

## Introduction

The diverse challenges faced in applying research principles across different societies, communities, and study types underscore the importance of taking contextual factors and social relations into account when conducting research.^1^ Community engagement in health research is considered one way of doing this,^2^ and employment of community members, often called field workers (FWs), is another.^3^ Given the lack of clarity in definitions and goals of community engagement, and the complex and contested nature of all of the key elements, employment of community members as research staff can be conceptualised as part of a wider set of community engagement activities, although there are important challenges with this, given the potential of fieldworkers to take on the interests of researchers as a result of financial or employment incentives.^4^

FWs are employed for both practical and ethical reasons, including supporting access to participants, addressing language barriers, advising on the culturally appropriate conduct of research, and minimising research costs.^5^ Overall it is recognized that FWs play a critical role in studies, in bridging language and cultural barriers, and in some cases in providing continuity in relations beyond the study period.^6^ However recent discussions on FWs have also highlighted some ethical dilemmas associated with their involvement in studies, including the potential for FWs to exploit community trust in their efforts to meet recruitment quotas, challenges in maintaining privacy and confidentiality in communities they are part of, and the possibility of FWs being exploited through unfair employment practices.^7^ More fundamentally, FWs may face tensions between professional expectations to adhere to ethical guidelines in the conduct of research, while simultaneously being responsive and sensitive to moral issues and dilemmas raised by members of their own communities.

We have argued elsewhere that the practical and ethical strengths and challenges that community-based staff or fieldworkers face is likely to differ depending on study type and on how embedded they are in the particular communities involved in that research.^8^ There can be a spectrum of employees, from those employed from local communities to continue to live in and work among those same communities, to those employed to live in a central place and work across large geographical areas. The more embedded fieldworkers are in a particular community, the more familiar they potentially are with local social networks and norms, with differing implications for the building up of appropriate trust relationships between research institutions and communities, and for the nature of support and supervision required.^9^ We have also argued that social relations established between FWs and research participants are key in research conduct; they can be facilitative of research ethics through creating environments of better discussion and understanding of the research, or undermine research ethics if those relations hinder or infringe on individual freedoms to make choices.^10^

In this paper we draw on a qualitative case study approach to explore how social relations between one group of fieldworkers and participants, and associated practical and ethical dilemmas, evolved and shifted over the course of the study. In so doing, we highlight the important but highly complex ethical work being conducted by frontline staff, and the impossibility of this work being monitored and supervised remotely.

### The case study and context

The KEMRI-Wellcome Trust Research Programme is a long-term, multidisciplinary, biomedical research centre mandated to carry out medical research in Kenya. All research carried out at the centre is reviewed by local, national scientific and independent ethical review committees, and where applicable external review committees. Additional mechanisms to strengthen communication and consent include a dedicated communication and consent committee, review and support of study specific community engagement plans, and locally designed informed consent templates.^11^

The programme has two main physical sites, the largest of which is in Kilifi, on the Coast, about 60 kms north of Mombasa, the second largest city in the country. The main activities and offices of KEMRI Kilifi are within Kilifi District Hospital (KDH), with many research activities conducted within the surrounding communities (approximately 250,000 residents) included in the centre's health and demographic surveillance system.^12^ The setting is characterised by very high levels of poverty, with strong power differentials within households based on age and gender.^13^

Of the approximately 800 staff employed in KEMRI-Kilifi, nearly a third are FWs. FWs in Kilifi are staff with at least 12 years of formal education, competitively recruited with formal contracts based on the programme's job grades and remuneration packages. As with most staff, FWs contracts are in line with study grant periods, usually with possibilities of extension on other studies. FW roles include information giving, performing biomedically ‘simple’ procedures such as preparing blood slides, collecting urine, and nasal samples, and filling out questionnaires. As will be seen later on in this paper, these activities are not necessarily perceived as risk free in communities, and are often far from ‘simple’ to perform in households. Programme support to FWs include training commensurate with skills required for their job, (for example communication skills and ethics), and supportive supervision.

We explored interactions between FWs and participants in a community based case study, purposively selected as it involved relatively high levels of interactivity between FWs and participants, and unfamiliar non-invasive study procedures (Box 1 summarises the ‘RSV-HH’ study). Data collection included participant observation of FW-participant interactions, group discussions with the ten study field workers (n = 3) and 16 household (HH) members (n = 5), and in-depth interviews (n = 3) with research staff. All interviews were conducted by DK, transcribed and translated into English. The thematic framework approach^14^ was used in analysis; data were organised and analysed in Nvivo 8.0. This paper discusses one key analytical theme: the development and evolvement of social relations over the course of the RSV-HH study, and implications of these relationships for fieldworkers’ dilemmas and challenges, and ultimately ethical practice.

**Box 1. Summary of case study: Respiratory Syncytial Virus household study (RSV-HH study)**

The RSV-HH study aimed to understand how RSV and other respiratory viruses are spread in the community and especially who infects infants in the households. This information would inform future vaccine strategies. Between October 2009 and June 2010, 50 households (HHs) in one locality within the research centre health and demographic surveillance system (HDSS), were recruited. Household eligibility included having an infant of less than 6 months at recruitment and at least one older sibling (<13 years); and consent from all HH members.

FW's formal roles in Study procedures: For all household members: temperature, a nasopharyngeal swab (NFS) and history of respiratory illness were taken every 3–4 days and saliva samples were taken once weekly. In addition for all children under 5 years a respiratory rate was taken at each visit. A demographic and risk assessment questionnaire was administered at the beginning and end of the study. Several data forms were filled for each participant at each visit.

Study risks: Mild discomfort during NFS taking and time inconvenience.

Benefits and compensation for participants: Free medical care for all common illnesses during study period; clinical visits to every participating household once a month at home. Also two chairs for each participating household, sweets for children and minors, educational materials to school going children, and in-kind token to each household at end of study.

Community benefits: boosting local health care facility through provision of drugs, additional clinical staff throughout the study period, and water treatment for all communal water points.

### Case study fieldworkers: who they are, formal roles, and amounts of interaction with participants

Ten field workers, 7 men and 3 women, were employed on the case study (Table 1).^15^ All were aged between 20 and 34 years old, and were given 9-month contracts. Although two FWs had previously worked for KEMRI in another short-term study, most had not had any formal employment before working for KEMRI. Most FWs (80%) were not married, and were born and brought up in rural Kilifi district, with the latter linked to an institutional policy to employ FWs from the location in which the study is being conducted where possible. Following 4 weeks of intensive training in communication skills and ethics, informed consent processes, and RSV-HH study protocol and procedures, the FW team had 1 month on-the-job training in the field including in information giving about the study and in taking the nasal swabs. Once the study started, support from the study team included regular interactions in the field with three senior FWs, a study coordinator and the study PI, all of whom commuted from Kilifi town to the study villages (approximately 20 minutes drive) on an almost daily basis throughout the data collection period.

**Table 1:** Socio-demographic characteristics of FWs in the study

Socio-demographic characteristic	Case study A FWs (n = 10) Number (%)
Gender (female)	3
Mean age, years (range)	26.5 (20–34)
<24 yrs	4
25–29	3
30–34	3
35–39	0
Marital status (married)	2
Education, average (range) years of schooling	12.2 (12–14)
12 years – O-level-	9
14 years – College/diploma	1
Average period (months) worked at KEMRI-WT	7.3 (5–9)
<=5 months	4
6–10 months	6
11–15 months	None
16–20 months	None
Number of FWs with relatives participating in the study	1
Contract period offered	9 months

Formal FW roles are summarized in Box 1 under study procedures. Each household visit took 1 to 4 hours, with much time spent responding to questions, re-confirming participation, and discussing study benefits. Discussions were not just about the study or KEMRI, but also current live topics in the community, for example the government allocation of community development funds.

Support to FWs included logistical support (office space, bicycles, back-packs, gloves, disinfectant and umbrellas), field-based supportive supervision, weekly field team meetings and peer-to-peer support. These formal and informal meetings were the settings in which many of the issues and challenges raised in this paper were discussed and, where possible, resolved. The whole research team also received support and advice on communication related dilemmas from a community engagement support group, as part of the broader community-engagement strengthening initiatives described above.

### Trajectory of building relationships: how social relations developed and evolved

**Figure 1:**
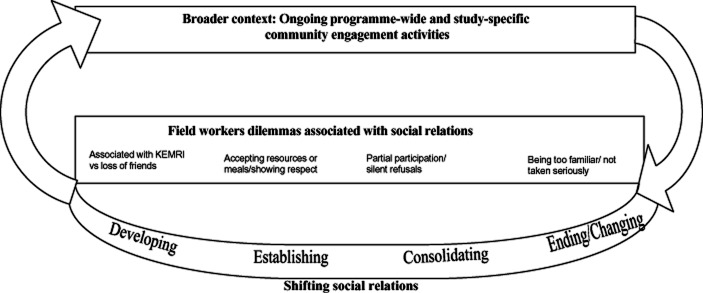
Evolving social relations and shifting dilemmas for fieldworkers in the RSV-HH study.

Social relations and dilemmas between participants and fieldworks were established, consolidated, and ultimately in some cases broken through the following four stages which broadly represented shifts in FW roles, and in FW-participant relationships and associated challenges and dilemmas; i) pre-entry consultation; ii) community entry and discussion of study procedures; iii) discussion of non-study procedures, and iv) exiting. Following an overview of these stages, the shifting dilemmas and challenges faced by FWs across these phases are discussed. The findings are summarised visually in Figure 1.

#### Pre-entry and community entry: developing and establishing relationships

All ten FWs came from and resided within the location where the study was conducted. As advised by community leaders and Ministry of Health, Community Health Workers (CHWs) introduced field workers to participating households within their jurisdiction at an initial household visit. Study researchers were particularly keen to collaborate with and work within established government and communal structures, to support study acceptability and to conform with locally recognised systems for community entry. Employing FWs from the community aimed to facilitate better follow-up of participating households, address cultural sensitivities around research conduct particularly with regards to interactions between FWs and household members. Employment of local residents is also regularly requested by community representatives, and is therefore encouraged in institutional policy as one form of ‘giving back’ to local communities, or of benefit-sharing.

Initial interactions at household level were reportedly particularly challenging to FWs; they were not fully confident in study procedures especially in collection of nasopharyngeal flocked swabs (NFS), and often faced difficulties in handling participants who were afraid of NFS. Gathering all household members twice a week (as per study protocols), and the considerable amount of time taken in each household (between 1 and 4 hours depending on the number of household members and the ease with which they participated in the study) added to the initial challenges FWs faced. Further pressure resulted from the urgency to recruit participants before the RSV epidemic season peaked. Initial hesitation and reluctance from potential participants was handled in various ways; including through discussing participants’ fears and concerns, going over consent information, checking participation decisions, reporting and requesting further clarification from supervisors and researchers, and allowing households time to consult over participation. The extent to which these approaches added pressure to participants to reconsider or even continue with the study was difficult to ascertain. Importantly however is that the consenting process was a negotiated one, involving significant persistence and patience among fieldworkers, and skills in knowing how to answer multiple questions and when to refer questions up the hierarchy to supervisors.

FWs reported that coming from the area, and being known in the community, helped facilitate initial acceptability of themselves and of the study.

FW2: …. my first interaction days with these households was not a joke because they thought I was coming from Kilifi, but it came to be an easier thing when they learnt I was coming from around. … *(Male, Exit FGD3)*

However being embedded in communities brought challenges; participants’ familiarity with FWs appeared to present further dilemmas, as discussed below.

#### Relationship building around and beyond study procedures: consolidating relationships

Complicating the development of initial relationships was participants’ dislike of the NFS. It was said to be uncomfortable and irritating to the nose. Reflex tears were mistaken for tears of pain and perceived as a sign of weakness, which was particularly problematic for male household members in this largely patriarchal community. What appeared to researchers to be a relatively simple non-invasive procedure, was therefore far from straightforward in practice, with participants perceiving it as uncomfortable, invasive and risky.

FWs’ frequent interactions with participants, including out-of-office hours and in other social spaces such as the dispensary or market place, provided opportunities to get to know one another, and to discuss FWs’ needs and pressures, and participant fears and concerns. Initial hesitation by some adult household members appeared to wane over time with participants increasingly accepting of the study procedures by the time of study exit. FWs talked of ‘continuous consenting’, where they continuously counterchecked participants’ research decisions especially where participants appeared hesitant to continue or disinclined to withdraw.

FW1:. … So what helped is that when you go there, you assume that they don't know anything at all [about this study], you start to consent them afresh. … When they see the importance of their participation and the situation that I face because of the way they are behaving [i.e. avoiding the FW]. … there are others who will sympathise with you, there are others who will be like okay he has told me something important, so let me give him the samples. (*Male, exit FGD2*).

Over time, and as hinted at above, there was a shift in interactions from that of formal professional to one infused with informality and relatedness. Familial titles such as daughter, son, grandchild, were used to describe the types of relationship that were evolving between FWs and participants in the negotiation of study procedures. Requests by participants for benefits and gifts beyond those officially provided by the study, such as for food items, cell phone airtime, and baby clothes, became increasingly common. FWs were sometimes also consulted on non-study related issues such as land ownership, planned community development projects and mentoring of young people. They were often referred to as doctors (*daktari*), partly as they work in KEMRI which is often seen as a hospital, but also because their work involved study procedures that are seen as doctors’ work, and as a sign of respect. These factors all contributed to some participants expecting FWs to diagnose illness and prescribe drugs.

FW5: …. They are really respectful because my friend here [references another FW] is always telling me he is being referred as a doctor there so (*All laughing*). … Yea, I think they look at me from a very serious perspective yea there is that mutual respect from there, am somebody important to them yea. (*Male, initial FGD1*)

FWs reported that being referred to as doctors offered them a status of respect, and an acknowledgement of their work. However, it is also possible that this created, at least in initial stages, a relationship akin to doctor-patient where participants may have felt limited in their ability to question or even challenge FWs views. While ‘kinship relations’ appeared to consolidate social relationships between participants and FWs (as exemplified by referring to each other in familial terms and associated kinship responsibilities; see also Geissler et al.^16^ ), the associated dilemma included a potential limitation on participants feeling able to express their views, if they differed with those of the FWs.

#### Exiting: changing of FWs and ending of study

The study employed four FWs (2 men, 2 women) mid-way to ease workload pressure on the other FWs. Initial challenges for the new FWs included not being readily accepted by some of the households that were handed over to them, largely as those households wanted to maintain positive relations already established with previous FWs. Being accepted by households mattered to the new FWs; it would ease their work, and facilitate their being accepted in the community and within their study team. A gradual handing over process was rolled out which included pairing of new and old FWs over 2–3 weeks and continued support post-handover. However, the new FWs reported being very distressed by households that appeared unwilling to work with them. They attributed this unwillingness to loyalty to the older group of FWs, and lack of confidence in the competence of the new group, as described by one of the researchers:

R2:. … So the participants once they are used to one fieldworker, any other fieldworker turning up at that home has a difficult time. They fail to trust us at all. And then even the young children, some of them shout at you that ‘*go and bring so and so that I can be swabbed*’, so it's really difficult for a fieldworker to be given another household to cover for another one. … (*female, initial IDI2*)

Strategies new FWs used to re-establish good relationships were similar to those earlier described at study entry including extended visits to households, often out-of-office hours, re-explaining the study, addressing fears and concerns and generally re-building trust.

Towards the end of the study, a careful study exit strategy (see Box 2) was rolled out which included reminders to the households sent several weeks in advance of study exit, a formal last visit by the study teams (researchers, clinician and FWs) to participating households, and a follow up visit two months post-study end to give aggregate study results and tokens of appreciation.

**Box 2. RSV HH-study exist strategy**

***Before end of study***

1. FWs reminded all household members, especially household heads, of study end; study clinician and researchers visited each household to inform on end of study and associated benefits.

***At end of study***

2. Study clinician or SFW accompanied FWs to formally bid goodbye to households and inform them of a later visit to feedback research results.
3. A meeting was held with community leaders where equipment purchased by the study was handed over to the dispensary committee and feedback of preliminary study results undertaken.

***After end of study***

4. About two months after the end of study, study researchers and remaining FWs visited each participant household; gave them a summary of preliminary results on study performance, a letter of appreciation, and gift hampers with a range of household items for each household.
5. FWs contract ended staggered based on time of entry and expiry of 9-months contract, following a send off party. Even though their contracts had ended, FWs continued to volunteer for about a month to another study of the same PI that involved schools; the study ended a month later. FWs appreciated this gradual exit as it allowed them to adjust to changes, from being employed to being without formal employment.
6. Two months later, four of the FWs were re-employed into the RSV-HH study, to assist in data entry. Later one of the FWs was employed in another study at the research centre following the Programme's recruitment process.

Of interest is that the end of the study, while important in reducing levels of interactions, did not always signify an end to relationships between FWs and participants. As one fieldworker explained:

FW3: … In fact they [participants] miss you so much, the moment they see you even on your way, on your own business they just request that even though you are through with your study you should find some time to just come and say hi, but don't forget us as such, they feel you are part of them. …”. (*Male, exit FGD3*).

### Field workers’ relationship dilemmas

The positive relations that developed through the intense and frequent interactions between FWs and participants were reportedly crucial to facilitating the study procedures and overall success of the study: 47 out of the target 50 households participated throughout the course of the study; and 8 dropped out (5 out-migrations and 3 refusals). But these relations also presented FWs with dilemmas and challenges.

#### Costing households but showing respect

The RSV-HH study began in the community at a time of little rain and food scarcity. Some households would offer a meal to FWs even though there was clearly not enough for household members. FWs agreeing to share a meal would show respect, and indicate acceptance into the family; declining a meal or compensating it (through cash) would be seen to be impolite. This situation presented dilemmas to FWs. Should FWs give something back in return? Who would pay for that? Would acceptance of food lead to families preparing relatively expensive meals? Would it undermine the FW's professional status? Might it lead to FWs exaggerating study benefits out of guilt? Would it take too much time-out of work for FWs and for participants? While there was no clear single solution, it was generally agreed among the trial team in peer groups and supervision meetings that FWs could politely decline a meal unless there were strong arguments in a particular case not to.

This dilemma illustrates the complexity on the ground of the balances between both tangible and intangible benefits and risks or disadvantages for participants.^17^ Appropriate approaches to benefit sharing during studies are critical to ethical practice, and are therefore typically reviewed as part of wider proposals by institutional and national ethics review committees, often with a focus on the potential of benefits to contribute to undue inducement. However, how these approaches unfold on the ground can be unexpected and difficult to predict in advance and therefore challenging to incorporate into initial plans.

#### Being employed by KEMRI: an achievement and a ‘sell-out’

There are a range of rumours and misperceptions about KEMRI-Kilifi, including for example that researchers practice devil worship.^18^ Similar rumours have been observed in other settings attributed to inadequate communication and resource differences between researchers and research communities.^19^ These rumours impacted on fieldworkers. For example, some non-participants reportedly accused FWs of having been inducted into devil worshipping activities, and spread word that they could not be trusted. FWs and participants were also sometimes taunted, mocked and given undermining nick-names. Beyond on-going negative rumours about the institution, fieldworkers attributed these behaviours to jealousy of them for having secured much desired employment, and jealousy of study participants for having gained access to study benefits. Questioning of FWs’ allegiance sometimes reportedly led to loss of friends:

FW4:. … I wasn't feeling well so I went to another kiosk to buy some painkillers … they saw [my work] T-shirt and then one shouted ‘you know what? The devil is here in the full swing’ (laughter). I told him I felt bad then and the rest laughed at me.” (*male, exit FGD3*).

These challenges illustrate the importance of employment practices in shaping the experience of fieldworkers for studies in contexts of poverty and inequity.^20^ Employment in these contexts can be highly contentious, with selection of inappropriate people or inequitable systems of selection potentially fuelling rumours and undermining consent processes, and having negative implications for broader relationships between communities and researchers. Once employed, how fieldworkers are supported to handle and respond to these challenges from community members is also important to the relationships so vital to ethical practice.

#### Partial participation: silent refusals, consent processes, and access to benefits

A popular Swahili saying quoted by FWs is ‘*Akufukizaye hakwambii toka*’ [the one who chases you away does not tell you ‘go']. This saying aptly captures the essence of silent refusals. ‘Silent refusals’ was used to refer to situations where participants participated inconsistently, or participated in some study procedures and not in others, without openly refusing or withdrawing from the study. A typical silent refusal scenario:

FW4:. … so he [the participant] is around the house, so when you tell him it is now his turn he tells you he is too busy, come back later. … And when you go and he tells you the time like noon or 1pm to be there, when you get there, there is no one, he has left. (*male, exit, FGD3*)

Over time, and through close interactions and familiarity with participants, FWs reported beginning to be able to identify silent refusals:

FW1:. … So its normal that it is not every time someone tells you ‘I don't want’, no, you have to learn them according to their actions or words then you completely know that this person does not want [to participate]. (*male, Exit, FGD2*)

Most silent refusals were in response to the NFS; and in many households at least one person disliked it. The dilemma was that dropping such silent refusals as per study protocol would have meant dropping the whole household, which would have lowered the study power. The study team were therefore often keen to have the refusal communicated verbally, to check that they had not misunderstood participants’ intentions. Such a request could have been perceived as pressurising participants against withdrawing, given that it required consultation with other household members. An alternative approach was to effectively ignore silent refusals, to allow for partial participation without openly discussing it. This approach was a mutually beneficial compromise that was often followed by the study team. For participants, it allowed the continued access to full benefits of the study since they did not withdraw; supporting their good relations within their households, and with FWs and the study team. For fieldworkers, they were able to allow those who they had developed relationship with, and who appeared to need to receive benefits to keep doing so. For the study, retaining silent refusals ‘quietly’ implied respecting the participant's decision not to withdraw (since this was not verbally communicated), and avoided encouraging further silent refusals.

There are clearly important implications of the concept of silent refusals and how they are handled by fieldworkers for consent processes and withdrawal, for benefit sharing during studies, for power relations between research participants and study teams, and for science. While a relatively legalistic approach to consent might require silent refusals to be identified and formally recognised, a moral commitment to autonomous individuals having an intrinsic right to make decisions about their bodies and lives^21^ might suggest the more nuanced approach taken by the study team is more appropriate. At the very least, this approach appeared to allow some participants to negotiate an involvement in the study on their own terms rather than in the black and white terms documented in study protocols.

#### From professional to personal relationships

A key feature of relations between FWs and participants was mutual respect; respect of cultural norms, and appropriate levels of respect to different household members and across different age-groups. While familiarity and informality enabled free discussions of issues, a high level of familiarity could also have led to a perception of lack of seriousness or even disrespect:

FW3: … sometimes you know that person who is too familiar with you, you may tell them something and they may treat it causally and may forget that professional part of you … and may treat you like a brother or sister; so when you go with the senior [researcher], they know that ‘ahh this is serious work and this issue that I'm being explained is not false, it is the truth’. … (*male, exit FGD2*)

Disrespectful behaviour was discerned in the way participants and FWs talked to each other, in interactions where the language used or the non verbal communication had sexual undertones (for example, behaviour between a man and woman considered inappropriate in that setting),or where FWs’ manner in a household was too casual. In some cases this disrespect was displayed by participants towards FWs. In FGDs, male FWs reported several instances; a young woman opened her blouse to a married male FW while he was taking her temperature; a married woman asked for a loan from a male FW and did not want her husband to know about it. Field workers expressed vulnerability in these situations; their handling of the situation could have led to loss of participants, marital conflicts, and a longer term issue in terms of FW and institutional reputations. Some FWs informed researchers of these situations, some sought advice from fellow FWs, others waited out for the end of study, and others still informed other household members in an effort to re-establish trust and respect.

Sexual undertones could be perceived as a sign of resistance towards FWs; an attempt to shame or embarrass them. It potentially leads to arguments between FWs and males in the household, and could even get FWs fired. It may have been a protest to the casualness of the relationship between some FWs and participants, or a challenge to the broader study or institution. Such interactions, though rare, illustrate the complex and constantly shifting power dynamics involved in research interactions in settings such as ours. As with silent refusals, they illustrate the potential for community members/participants to influence studies and relationships in ways that are not immediately obvious in what are sometimes strongly inequitable research contexts.

## Conclusion

Social relations, although rarely discussed in research conduct, form the fabric in which the dialogue, information sharing and negotiations that are central to ethical practice take place. Even in this relatively small case study, we see how the relationships between fieldworkers and study participants were complex, and ever-shifting, and had implications for consent, access to benefits and mutual understanding and trust. The interactions between FWs and participants were influenced by the type of study (repeat visits to entire households), and by the broader community-institution relationships to which they also contributed. In this study, FWs were socially embedded in the study population as they were residents of the study population. FWs were aware of and advised on cultural sensitivities of research, including language and norms of interactions with community members. While familiarity and friendship created an environment for mutual understanding through continuous dialogue over the course of the research, paradoxically they also posed relationship-related dilemmas with potential to undermine the research ethics FWs were employed, at least in part, to promote. Dilemmas included difficulties for participants in expressing dissenting opinions of the study, relatedness costs to FWs, and muddling between professional behaviour and casual conduct of the FWs. These findings emphasise the importance of supportive supervision for field workers throughout studies, including support for handling unexpected questions and situations, and encouragement to share dilemmas faced and how to best resolve these. While such support is essential for ethical practice in the field it is unlikely to ever be able to prevent or solve the dilemmas entirely.
